# White Grape Cell Walls as Fining Agents in Red Wine: Mechanistic Insights from ATR-FTIR Spectroscopy

**DOI:** 10.3390/foods15061050

**Published:** 2026-03-17

**Authors:** Julia Gómez-Pérez, Berta Baca-Bocanegra, José Miguel Hernández-Hierro, Krzysztof B. Beć, Christian Wolfgang Huck, Julio Nogales-Bueno

**Affiliations:** 1 Food Colour and Quality Laboratory, Área de Nutrición y Bromatología, Facultad de Farmacia, Universidad de Sevilla, 41012 Sevilla, Spain; jgomez11@us.es; 2 Department of Analytical Chemistry, Facultad de Farmacia, Universidad de Sevilla, 41012 Sevilla, Spain; bbaca1@us.es (B.B.-B.); julionogales@us.es (J.N.-B.); 3 Institute of Analytical Chemistry and Radiochemistry, University of Innsbruck, Innrain 80-82, 6020 Innsbruck, Austria; krzysztof.bec@uibk.ac.at (K.B.B.); christian.w.huck@uibk.ac.at (C.W.H.)

**Keywords:** ATR-FTIR, grape pomace, cell wall material, wine, sediment, fining

## Abstract

The fining of red wines is a crucial process for enhancing their sensory quality, involving the elimination of compounds that compromise stability or generate undesirable attributes. Against the backdrop of growing interest in sustainable and allergen-free alternatives, this study examines the potential of using cell wall material from white grape pomace (Zalema, Pedro Ximénez and Moscatel varieties) as fining agents in red wine. Cell wall samples were isolated and characterised using attenuated total reflectance Fourier-transform infrared spectroscopy (ATR-FTIR). PCA applied to the cell wall spectra revealed homogeneous matrices dominated by structural polysaccharides, including cellulose, hemicellulose, lignin, pectins and arabinogalactans. Following the fining treatments, significant differences were observed in the generated sediments compared to the control, primarily due to signals attributable to phenols, proteins, and carbohydrates. This demonstrates the active involvement of these compounds in the formation of precipitates. The results confirm that the composition of the cell wall favours interaction with phenols and proteins in wine, thereby contributing to their elimination. Overall, this work demonstrates the potential of cell wall material from white grape pomace as plant-based oenological fining agents, providing new insights into the molecular mechanisms of action.

## 1. Introduction

Fining is one of the most widely used winemaking techniques for improving the stability and sensory quality of wine [1,2,3,4]. The purpose of fining is to remove or reduce components that, when present at high levels, may compromise clarity, cause colloidal instability or intensify negative attributes such as astringency and bitterness [5,6,7]. The process involves adding a substance capable of interacting with the target compounds typically through adsorption, aggregation, or the formation of insoluble complexes which are subsequently removed by sedimentation or filtration [4,8,9]. The components that most frequently justify the use of fining treatments are flavanols and other families of phenolic compounds. At elevated concentrations, these can alter the gustatory balance of the wine [10].

Historically, the most commonly used fining agents have been animal-derived proteins such as gelatine, egg albumin, caseinates and isinglass, due to their strong affinity for condensed tannins. However, their use presents several challenges: they can trigger allergic reactions in sensitive consumers, they are subject to strict labelling regulations, and they are not suitable for vegetarians or vegans [1,11,12]. These limitations have led to the development of plant-based alternatives, including proteins extracted from cereals, legumes, tubers, algae and grape seeds [1,3,11,13,14,15].

Growing interest in sustainable winemaking practices and the need to reduce reliance on allergenic agents has led to research into alternative materials derived from winery by-products. In this context, grape pomace, the main solid residue generated during winemaking, emerges as a particularly valuable resource [16,17,18,19]. Among the different types of grape pomace, white grape pomace is produced in large quantities and is a particularly promising material, as it is not subjected to maceration or fermentation during wine production in most cases. Therefore, it retains a high concentration of bioactive compounds. Furthermore, as white grape pomace is the first by-product generated during the harvest season, it is readily available for use in the fining of red wines [20,21,22,23].

Due to the significant quantity of pomace produced globally, its utilisation has become a key objective of European policies seeking to reduce waste and enhance efficiency throughout the agri-food sector. In this context, the revalorisation of agri-food by-products such as grape pomace is closely aligned with United Nations Sustainable Development Goal 12, which promotes the reduction in waste generation through prevention, recycling and reuse [24]. Composed of skins, seeds and stems, pomace contains a matrix rich in structural polysaccharides which, according to recent studies, can interact with phenolic compounds in wine [25,26]. Previous research has demonstrated that grape and pomace cell walls can reduce phenolic content and decrease turbidity, suggesting their potential use as natural fining agents. The initial studies that focused specifically on fining with cell wall fractions reported significant reductions in phenolic composition, achieving efficiencies comparable to those of certain commercial fining agents [18,27,28].

As well as their potential as alternative fining materials, grape cell walls fully align with a circular economy approach, as they enable the recovery of value from an abundant and widely available winery by-product. However, grape pomace is intrinsically heterogeneous. Its composition may vary considerably depending on grape variety, degree of ripeness, vineyard and climatic conditions, vinification practices, and the relative proportions of skins, seeds, and stems present in the pomace. These factors directly influence the polysaccharide profile, lignin content, phenolic composition, and structural organisation of the cell wall matrix [29,30,31,32]. This natural variability poses significant experimental challenges, particularly in terms of reproducibility, extraction yield, structural characterisation, and the interpretation of technological behaviour. Differences in compositional and structural features may lead to variability in the interaction capacity of cell wall materials with wine phenolic compounds, thereby affecting their fining performance.

To address this variability, a spectroscopic screening strategy was previously applied for sample selection, allowing the classification of samples according to their spectral characteristics and ensuring the representativeness of the subset analysed in the present study [23,33,34].

In parallel, the authors have proposed a fining index to evaluate the performance of novel fining agents. This index integrates the reductions observed in the main phenolic families affected during wine fining and was applied to the samples selected with NIR mentioned above. This index enables emerging alternatives to be compared with well-established commercial clarifying agents and provides a valuable means of predicting their performance prior to larger-scale trials [27]. Although different analytical approaches have been used to study fining mechanisms in wine, including phenolic profiling and particle stability analyses, the structural characterisation of the sediment fraction remains limited [35].

The aim of this study is therefore to investigate the fining mechanism in red wine using cell wall material extracted from the pomaces of different varieties of white grape through attenuated total reflectance Fourier-transform infrared (ATR-FTIR) spectroscopy. Mid-infrared spectra, commonly acquired in Fourier-transform (FT) mode, display narrow and well-defined absorption bands that correspond to fundamental molecular vibrations and can be directly assigned to specific chemical structures [36]. This characteristic makes FTIR spectroscopy particularly suitable for the detailed characterisation of complex matrices such as grape cell walls and wine sediments, as it allows the identification of the main functional groups involved in intermolecular interactions. In this study, ATR-FTIR provides valuable information on the structural and chemical changes that occur during the fining process, offering deeper insight into the mechanisms governing the interactions between cell wall materials and wine components. This knowledge supports the development of sustainable and efficient alternatives for the wine industry.

## 2. Materials and Methods

### 2.1. Samples

#### 2.1.1. Cell Wall Material from White Grape Pomace

A total of fifteen cell wall samples were selected from white grape pomace native to Andalusia (Zalema (*n* = 3), Moscatel (*n* = 6) and Pedro Ximénez (*n* = 6)) originating from an initial set of 111 white grape pomace samples collected from different wineries and used for the preliminary spectroscopic screening as described in detail in Gómez-Pérez et al. [23]. To carry out this selection, the spectral data for each sample were recorded using a Micro-NIR Pro Lite 1700 device (VIAVI, Santa Rosa, CA, USA) for near-infrared spectroscopy, following the procedure described by [23]. To preserve the highest possible spectral diversity, principal component analysis (PCA) was applied to the dataset and Mahalanobis distances (H) were calculated. Based on a similarity threshold of NH ≤ 0.6, eleven groups were identified, and one representative sample was selected from each group. These eleven samples, together with four spectral outliers, constituted the set of fifteen samples used in this study.

Extraction of the cell wall material (CWM) was carried out using adapted methods [26,37,38]. Briefly, 10 g of grape skins were subjected to a short heat treatment and subsequently homogenised. The resulting material was mixed with ethanol and treated with ultrasound to remove soluble compounds. The alcohol-insoluble solids (AIS) were then recovered by filtration. The AIS were repeatedly washed with hydroalcoholic solutions to confirm the absence of sugars in the resulting liquids (supernatants) using the [39] test. They were then rinsed with ethanol and acetone, dried and ground to obtain a homogeneous powder. This powder was used as the fining agent in this study [23].

#### 2.1.2. Sediment After Fining Red Wines

A total of sixteen sediment samples were obtained from fining assays performed on a red wine. Fifteen of these sediments corresponded to wines fined with each of the previously extracted cell wall materials. One additional sediment originated from a control sample consisting of a wine that was not fined with any agent. The assays were carried out in triplicate using 200 mL glass vessels. The cell wall materials were added at a dose of 0.1 g/L, which was selected based on previous experimental assays [23,27], mixed with the wine and stored in the dark at 25 ± 2 °C for six days. Control samples were prepared under the same conditions. After the fining period, the wines were separated from their lees (i.e., sediment samples), which were collected for analysis.

Finally, all sediments were freeze-dried to yield dry powders suitable for ATR-FTIR analysis, thus constituting the final set of sixteen sediment samples (in triplicate) evaluated in this study.

### 2.2. ATR-FTIR Data Collection

ATR-FTIR spectra were acquired using a PerkinElmer Spectrum 400 FT-IR spectrometer (PerkinElmer Inc., Waltham, MA, USA) equipped with a diamond ATR sampling accessory and controlled by Spectrum software (version 6.3.1). Prior to each measurement sequence, a background spectrum was collected under the same instrumental conditions and automatically subtracted by the software during spectral acquisition.

The samples, initially as dry powders, were homogenised manually to ensure uniformity. From each homogenised batch, three independent aliquots were taken to evaluate measurement repeatability. Each aliquot was placed directly onto the diamond crystal and pressed with a constant force of 150 internal pressure units to ensure proper contact with the ATR surface.

Spectra were recorded over the 4000–650 cm^−1^ wavenumber range at a spectral resolution of 4 cm^−1^. For every aliquot, 16 scans were accumulated and averaged to improve the signal to noise ratio. All measurements were performed at room temperature (approximately 22 °C).

### 2.3. Data Analysis

All data analyses were performed using The Unscrambler X 10.4 (64 bit) software (Camo, Oslo, Norway). Prior to any pre-processing, the spectra were converted to pseudoabsorbance. ATR-FTIR spectra were then evaluated using two scatter-correction methods-standard normal variate (SNV) and multiplicative scatter correction (MSC) [40,41]. Both approaches produced comparable results, with no appreciable differences in the reduction in scattering effects or overall spectral quality. Because of this similarity and the simpler implementation of SNV, it was selected as the pre-processing method for the ATR-FTIR dataset.

Following SNV correction, for comparative purposes, average spectra were calculated for each sample type, namely the cell wall extracts, the untreated control sediment, and the sediments obtained after the fining treatments. Since SNV alone provided consistent and stable spectra across all measurements, no derivative transformations or baseline corrections were required.

After pre-processing, an initial joint principal component analysis (PCA) was performed including all sample types. This preliminary model revealed a clear separation between the two main types of samples (cell wall materials and sediments), confirming the existence of distinct spectral profiles and, consequently, different chemical compositions (Figure S1). However, this strong differentiation between sample categories could potentially mask the intrinsic variability within each group. Therefore, separate PCA models were subsequently developed to allow a more detailed interpretation of the compositional variations within each sample type.

In total, two main PCA models were considered for interpretation. The first PCA included only the cell wall samples from the three white grape varieties—Moscatel, Zalema, and Pedro Ximénez—to assess potential structural differences between them. The second PCA considered two sample categories (control sediment and sediments) to evaluate the spectral differences between them and to gain insight into the mechanism of action of the cell wall material during the fining process, particularly through the compositional changes reflected in the sediments.

PCA is an unsupervised pattern-recognition technique used to explore the latent structure of spectral datasets. In exploratory analyses, it is common not to rigidly define the number of components retained, as the objective is to identify the most relevant sources of variance within the data. In this study, seven PCs were obtained and the first two principal components (PC1 and PC2) were selected, as they accounted for the highest proportion of spectral variability. The interpretation of their associated loading plots enabled the identification of the characteristic vibrational bands and peak regions responsible for the separation of samples in the score plots and, in this context, provided valuable information on the chemical groups involved in the interactions between the cell wall material and wine components during fining.

## 3. Results and Discussion

### 3.1. Spectral Characteristics of the Samples

**Figure 1:**
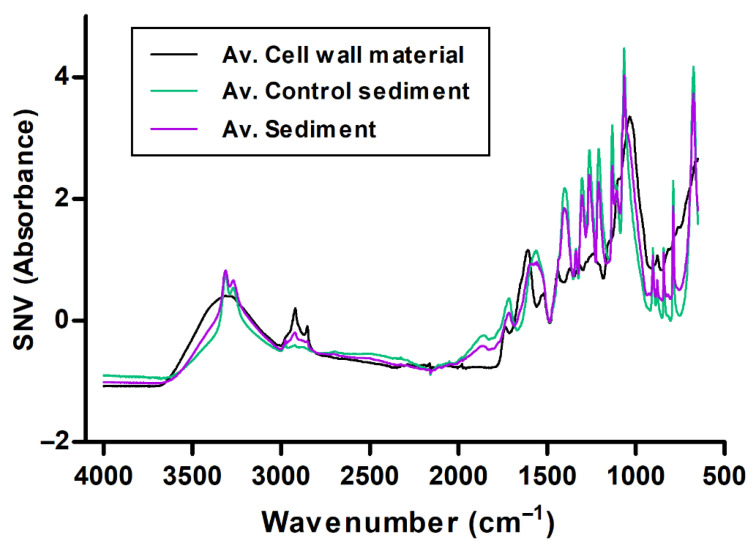
Averages of the spectra of the cell wall material from white grape pomace, sediment, and control sediment from the samples.

Figure 1 shows the mean spectra obtained for each sample type after SNV pre-treatment. At first glance, clear differences can be observed between the spectra corresponding to sediment and cell wall samples, particularly in two well-defined spectral regions. The first region, between 3304 and 2975.5 cm^−1^, is associated with O-H and C-H stretching vibrations. The second region, ranging from 1440 to 1100 cm^−1^, is related to vibrational modes that are characteristic of polysaccharides and other cell wall structural components.

These spectral differences suggest distinct chemical compositions in the two types of samples. To confirm this, a joint principal component analysis (PCA) was performed, which revealed a clear separation between the two types of samples (Figure S1). However, given that this strong differentiation could mask the intrinsic variability within each sample type, separate PCA models were subsequently developed: one for the cell wall samples and another for the sediment samples. This allows for a more detailed interpretation of the compositional variations within each group.

### 3.2. ATR-FTIR Analysis of Cell Wall Material from White Grape

**Figure 2:**
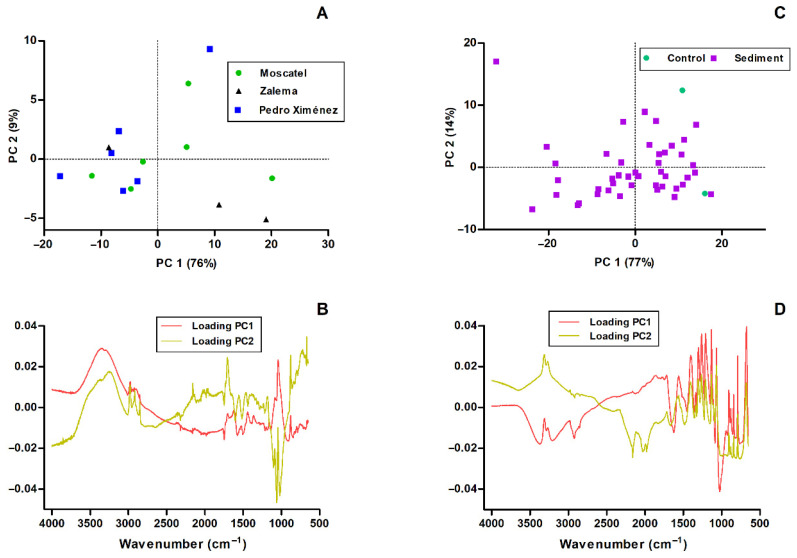
Score plots of the first two principal components after performing PCA on the ATR-FTIR spectra recorded from samples of cell wall material from white grape pomace (**A**) and the sediment obtained after the fining of wine (sediment with those cell walls and control sediment) (**C**). Loading plots of PC1 and PC2 for the cell wall materials derived from white grape pomace (**B**) and for the fined wine sediments and control wine sediments (**D**).

Principal component analysis (PCA) applied to the ATR-FTIR spectra of the cell wall samples enabled the identification of structural differences among the Moscatel, Zalema, and Pedro Ximénez varieties, as well as the vibrational bands responsible for this variability. Figure 2A shows the scores of the cell wall materials from white grape pomace in the space defined by the first two principal components (PC1 and PC2), which individually explain 76% and 9% of the total spectral variance, respectively. The three studied varieties are homogeneously distributed in this space, indicating a higher structural heterogeneity in their cell wall composition. However, some slight trends can be ventured, such as that most Pedro Ximénez samples have negative scores for PC1 and that most Zalema samples have scores in the second quadrant.

The loading plots (Figure 2B) were interpreted to reveal that PC1 is dominated by bands closely related to the polysaccharide and phenolic fractions of the cell walls. Significant contributions were observed in the 3300–3250 cm^−1^ region, which corresponds to the stretching of the O-H bonds of the phenolic groups, and in the 2980–2850 cm^−1^ region, which is associated with aliphatic C–H stretching vibrations (CH_2_ and CH_3_), commonly present in organic constituents of the cell wall materials used as fining agents [42,43]. A significant contribution was also observed at 1746 cm^−1^, which corresponds, according to the literature, to the region around 1736 cm^−1^, assigned to the C=O stretching vibrations of esterified uronic acids [44]. Variations in this spectral region indicate differences in the degree of esterification (DE) of pectins, suggesting varietal differences in the esterification level of the pectic fraction. Lower DE increases the proportion of free carboxyl groups, directly affecting pectin reactivity and its ability to interact with phenolic compounds during wine fining [45,46,47,48,49,50,51].

PC1 includes contributions from the 1700–1580 cm^−1^ region, which correspond to the vibrations of C=O and COO^−^ in carboxylic acids, esters, carboxylates, and proteins [42,52,53]. This reflects differences in pectin structure and other matrix components. Contributions at 1237–1157 cm^−1^ and 1140–920 cm^−1^ correspond to vibrations of C-O, O-H and C-O-C, which are typical of cellulose, hemicellulose, lignin, pectins and arabinogalactans. This indicates differences in polysaccharide architecture [50,51,54,55,56,57,58,59]. For a clearer visualisation of the spectral assignments discussed above, these have been summarised in Table 1.

Although most of the spectral characteristics contributing to PC2 have already been discussed in PC1, there are still some characteristics that need to be mentioned. In general, PC2 explains the more subtle variations relating to the aromatic and lignified components of the cell walls. The bands at 1570–1504 cm^−1^ and 1480–1385 cm^−1^ correspond to aromatic skeletal vibrations and C=C stretching, which are associated with lignin and phenolic compounds [43,47,60]. Meanwhile, the region at 900–670 cm^−1^ reflects the out-of-plane C-H bending that is typical of aromatic rings [43].

**Table 1:** Main functional groups to the different vibrations present in the ATR-FTIR spectra of cell wall material from white grape pomace.

Absorption Bands (cm^−1^)	Assignment	Component	References
**3300–3250**	ν (O-H)	Phenols	[42]
**2980–2850**	ν (CH_3_), ν (CH_2_)	Aliphatic groups in organic compounds	[42,43]
**1736**	ν (C=O)	Esterified uronic acid	[44]
**1700–1580**	ν (C=O), ν_as,s_ (COO)	Carboxylic acid, carboxylate, ester, protein	[42,52,53]
**1570–1504**	ν (O-H), ν (C=C), Benzene ring skeleton vibration ν (C–C) aromatic	Aromatic compounds, lignin	[47,61]
**1480–1385**	C=C-C ^a^	Aromatic ring stretch	[43]
**1370**	δ (C–H)	Cellulose, hemicellulose	[54,55,56,57]
**1237**	ν (C-O), δ (O-H)	Polysaccharides, pectins, lignins	[50,51,58]
**1157**	ν (C–O–C)	Cellulose, hemicellulose	[54,55,56,57]
**1140–920**		Arabinogalactan, cellulose	[54,55,56,57,59]
**900–670**	(C-H)	Aromatic out-of-plane bend	[43]

^a^ used as an approximation of the unique aromatic ring bonding.

Overall, the results do not show any significant differentiation; the slight differences are mainly due to variations in structural polysaccharides, such as pectins, cellulose, hemicellulose and arabinogalactans, as well as the relative abundance of phenolic residues and lignin. It can also be observed that grape variety does not play a dominant role in the distribution pattern of the cell wall materials obtained from heterogeneous samples. Instead, the differentiation appears to be more strongly associated with their specific structural and compositional characteristics rather than varietal origin. These compositional differences are likely to influence the fining behaviour of each cell wall material, thereby affecting their capacity to interact with wine phenolic compounds during the clarification process.

### 3.3. ATR-FTIR Analysis of Control and Treated Wine Sediments

Principal component analysis (PCA) was applied to the ATR-FTIR spectra of control sediment and sediment samples in order to evaluate the compositional changes associated with the fining process. The model explained 77% of the total variance in PC1 and 14% in PC2, indicating that the main chemical differences between the two sample types are effectively described by the first two components (with a total of 91% of cumulative variance). In the score plot (Figure 2C), a small trend between control sediment and sediment samples is observed along PC1, reflecting the substantial chemical modifications induced by fining.

Sediment samples are distributed across a wide area of the score plot, whereas control sediment samples are mainly located in the positive region of PC1. This distribution indicates that PC1 captures the compositional differences between soluble wine components and the more heterogeneous insoluble material forming the sediment fraction. The interpretation of this separation is supported by the loading plot (Figure 2D), which reveals that PC1 is dominated by spectral regions associated with phenolic compounds, polyols, and carbohydrates.

Specifically, strong contributions from the 3375–3210 cm^−1^ region, attributed to O-H stretching vibrations of phenolic groups, and in the 2980–2850 cm^−1^ region, which is associated with aliphatic C–H stretching vibrations (CH_2_ and CH_3_), commonly present in organic constituents of the sediment matrix, such as tartrate salts, phenolic compounds, and residues of the cell wall materials used as fining agents [42,43]. Additional relevant contributions at 1716 cm^−1^, assigned to C=O stretching of esters and organic acids [44], and at 1621 cm^−1^, associated with COO^−^ vibrations of carboxylates, suggest the presence of polysaccharide-derived fragments and modified organic acids within the precipitated material [42].

Further differentiation is observed in the 1480–1355 cm^−1^ region, related to aromatic C=C stretching, which reflects the incorporation of aromatic structures such as tannins into the sediment phase [43]. In contrast, control sediment samples show a greater contribution from bands associated with alcohols and carbohydrates, particularly in the 1086–923 cm^−1^ region [62], as well as aromatic C-H out-of-plane bending vibrations in the 900–670 cm^−1^ range [43]. These features are characteristic of soluble wine components and are less pronounced in the sediment spectra (Figure 1). For a clearer visualisation of the main spectral assignments discussed above, these have been summarised in Table 2.

Although PC2 accounts for a smaller proportion of variance, it highlights additional differences related to nitrogen-containing compounds. The contribution of the amide-related band at 1565 cm^−1^ suggests the involvement of proteins in the sedimentation process, probably through protein–phenolic interactions that contribute to aggregate formation and precipitation [63].

**Table 2:** Main functional groups assigned to the different vibrations present in the ATR-FTIR spectra of sediments.

Absorption Bands (cm^−1^)	Assignment	Component	References
**3375–3210**	ν (O-H)	Phenols	[42]
**2985–2855**	ν (CH_3_), ν (CH_2_)	Aliphatic groups in organic compounds	[42,43]
**1994–1779**	“Combi” ^b^	Aromatic combination bands	[43]
**1716**	ν (C=O)	Esters, organic carboxylic acids, aldehydes	[42]
**1621**	ν_as,s_ (COO)	Carboxylic acid, carboxylate, ester	[42]
**1565**	δ (N-H), ν (C-N)	Secondary amide	[63]
**1480–1355**	C=C-C ^a^	Aromatic ring stretch	[43]
**1335**	δ (C-H), ν (CH_2_)		[42]
**1302**	(C-N), (O-H)	Aromatic primary or secondary amine, CN stretch Primary or secondary, OH in-plane bend	[43]
**1260–1209**	ν (O-H), δ_in-plane_ (C-H)	Aromatic compounds and their derivatives	[42]
**1131**	ν (C-O)	Phenols	[42]
**1086–923**	ν (C-O)	Primary alcohol or carbohydrates	[62]
**900–670**	(C-H)	Aromatic C-H out-of-plane bend	[43]

Assignment: *v* stretching; *δ* bending. ^a^ used as an approximation of the unique aromatic ring bonding. ^b^ “Combi” denotes assignment to combination bands.

Overall, Figure 2C,D demonstrate that the fining process leads to the selective removal of phenolic compounds, carbohydrates, and proteins from the wine matrix, resulting in sediments enriched in these components. The clear spectral distinction between control sediment and sediments supports a fining mechanism based on the formation of insoluble aggregates driven by phenolic–polysaccharide and phenolic–protein interactions, ultimately contributing to the improvement of wine clarity and colloidal stability.

### 3.4. Proposed Mechanism of Cell Wall Action During Wine Fining

The combined analysis of the mean spectra and PCA results provides a coherent interpretation of the mechanism by which grape cell wall material participates in wine fining. The ATR-FTIR spectra of sediment samples show enrichment in bands associated with phenolic compounds, esters, polysaccharide fragments, and amide signals, indicating that fining involves a complex set of interactions among wine phenolics, polysaccharides, and proteins.

Cell wall material from white grapes pomace contains a matrix rich in pectins, hemicelluloses, cellulose, and arabinogalactans, whose functional groups—primarily OH, C-O-C and COO^−^—serve as interaction sites for wine phenolics, particularly tannins and aromatic structures. The band near 1740 cm^−1^, associated with the C=O stretching of esterified uronic acids, and its variation among varieties, suggests that the degree of esterification (DE) of pectins is a key parameter: lower DE increases the proportion of negatively charged COO^−^ groups, enhancing the ability of pectins to bind phenolic compounds.

The appearance of ester and carboxylate bands (1716 and 1621 cm^−1^) in sediment spectra suggests that modified pectin fragments and other polysaccharides co-precipitate with phenolics. Along with enhanced signals in carbohydrate-related regions (1086–923 cm^−1^), this indicates that fining involves not only adsorption but also co-aggregation and co-precipitation of structural polysaccharides.

Additionally, the amide-related band at 1565 cm^−1^ in sediments suggests that unstable wine proteins are also retained, likely through hydrogen bonding or hydrophobic interactions with phenolics previously adsorbed onto the cell wall surfaces. This supports the observed improvement in protein and colloidal stability after fining.

Together, these findings support the following mechanism [4]:

1. Initial interaction: OH and COO^−^ groups of cell wall polysaccharides form hydrogen bonds, ionic interactions, and hydrophobic associations with wine phenolics.
2. Complex formation: low-DE pectins (1740 cm^−1^) promote aggregation with phenolics, forming larger macromolecular complexes.
3. Co-precipitation: polysaccharide fragments and denatured proteins become incorporated into the aggregates.
4. Final sedimentation: insoluble complexes precipitate, leading to sediments enriched in phenolics, modified pectins, and proteins—consistent with spectral and PCA observations.

This mechanism explains the reduction in phenolic compounds associated with astringency, colour and bitterness, as well as improvements in turbidity and colloidal stability following fining with cell wall material. A better understanding of the fining mechanism may contribute to the optimisation of this process, promoting the selective precipitation of undesirable components while limiting the removal of desirable compounds.

## 4. Conclusions

ATR-FTIR spectroscopy combined with multivariate analysis confirmed that the cell wall material extracted from grape pomace exhibits a complex structural matrix dominated by lignin, cellulose, hemicellulose, pectins, and arabinogalactans. The first principal component (PC1) included strong characteristics revealing differences on the relative proportions of phenolics, polyols, and structural polysaccharides, indicating that compositional variability among cell walls directly influences their fining behaviour.

Analysis of the sediment fraction revealed that samples obtained after fining with cell walls were enriched in phenolic compounds, carbohydrates and proteins, consistent with the dominant O-H, C-H and COO^−^ bands observed in the loading plots. Compared to the control sediment, sediments from cell-wall-treated wines displayed increased intensities in these regions, suggesting that interactions between cell wall polysaccharides and wine constituents promote the formation of insoluble complexes that eventually precipitate (Figure 1).

These findings support a fining mechanism in which initial adsorption of phenolics and proteins onto the cell wall matrix is followed by the formation of aggregates and subsequent sedimentation. The role of pectins—particularly their degree of esterification—together with contributions from hemicelluloses and other polysaccharides, appears to be central to the effectiveness of the process.

Overall, this study demonstrates that grape pomace cell walls represent a promising natural alternative to conventional fining agents, capable of reducing compounds associated with astringency, turbidity and colloidal instability. Their use also aligns with circular-economy principles by valorising a major winery by-product. These results open new opportunities for the development of plant-based fining materials that enhance both sustainability and selectivity in wine fining.

## Data Availability

The original contributions presented in this study are included in the article. Further inquiries can be directed to the corresponding author.

## Supplementary Materials

The following supporting information can be downloaded at: https://www.mdpi.com/article/10.3390/foods15061050/s1.
