# Supplementary material for: White Grape Cell Walls as Fining Agents in Red Wine: Mechanistic Insights from ATR-FTIR Spectroscopy
Source: Foods. 2026 Mar 17;15(6):1050. doi: 10.3390/foods15061050 (PMC13025305; doi:10.3390/foods15061050)
Supplement: Supplementary file 1 [file foods-15-01050-s001.zip › foods-4197546-supplementary.pdf]

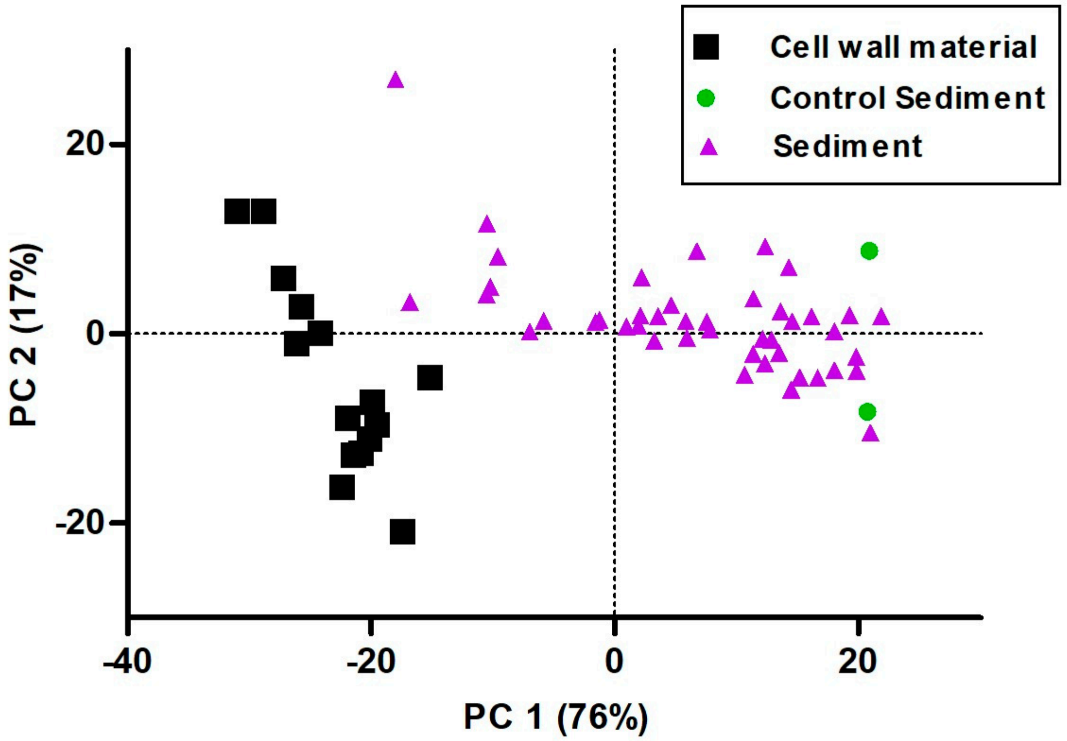

Figure S1. Score plots of the first two principal components after performing PCA on the ATR-FTIR spectra recorded from samples of cell wall material from white grape pomace, and the sediment obtained after the fining of wine (sediment with those cell walls and control sediment).
